# Supplementary figures and images for: Exploring the Molecular Mechanism of Skeletal Muscle Development in Ningxiang Pig by Weighted Gene Co-Expression Network Analysis
Source: Int J Mol Sci. 2024 Aug 22;25(16):9089. doi: 10.3390/ijms25169089 (PMC11354759; doi:10.3390/ijms25169089)

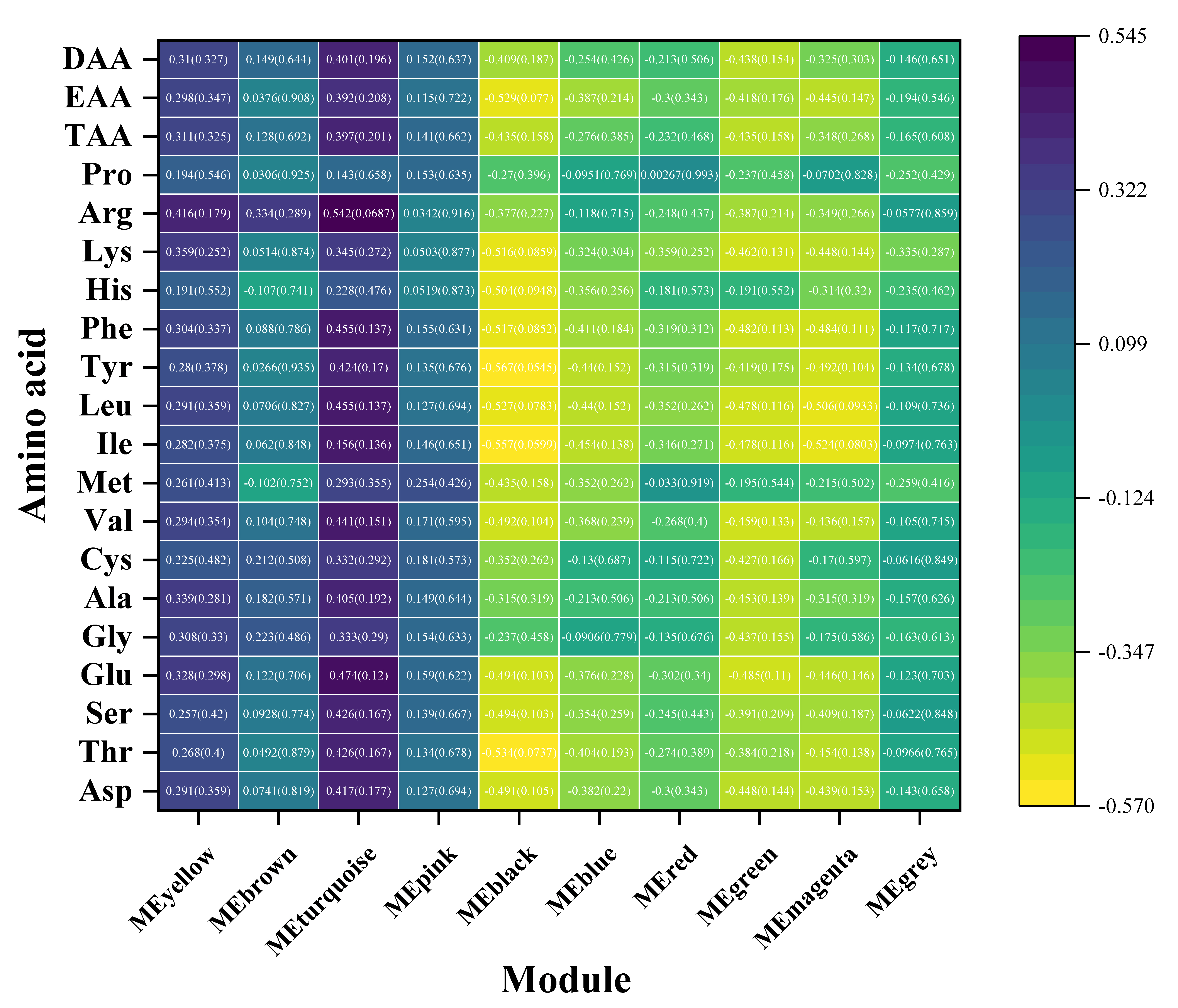

Supplement: Supplementary file 1 [file ijms-25-09089-s001.zip › ijms-3117345-supplementary/Figure S1.jpg]

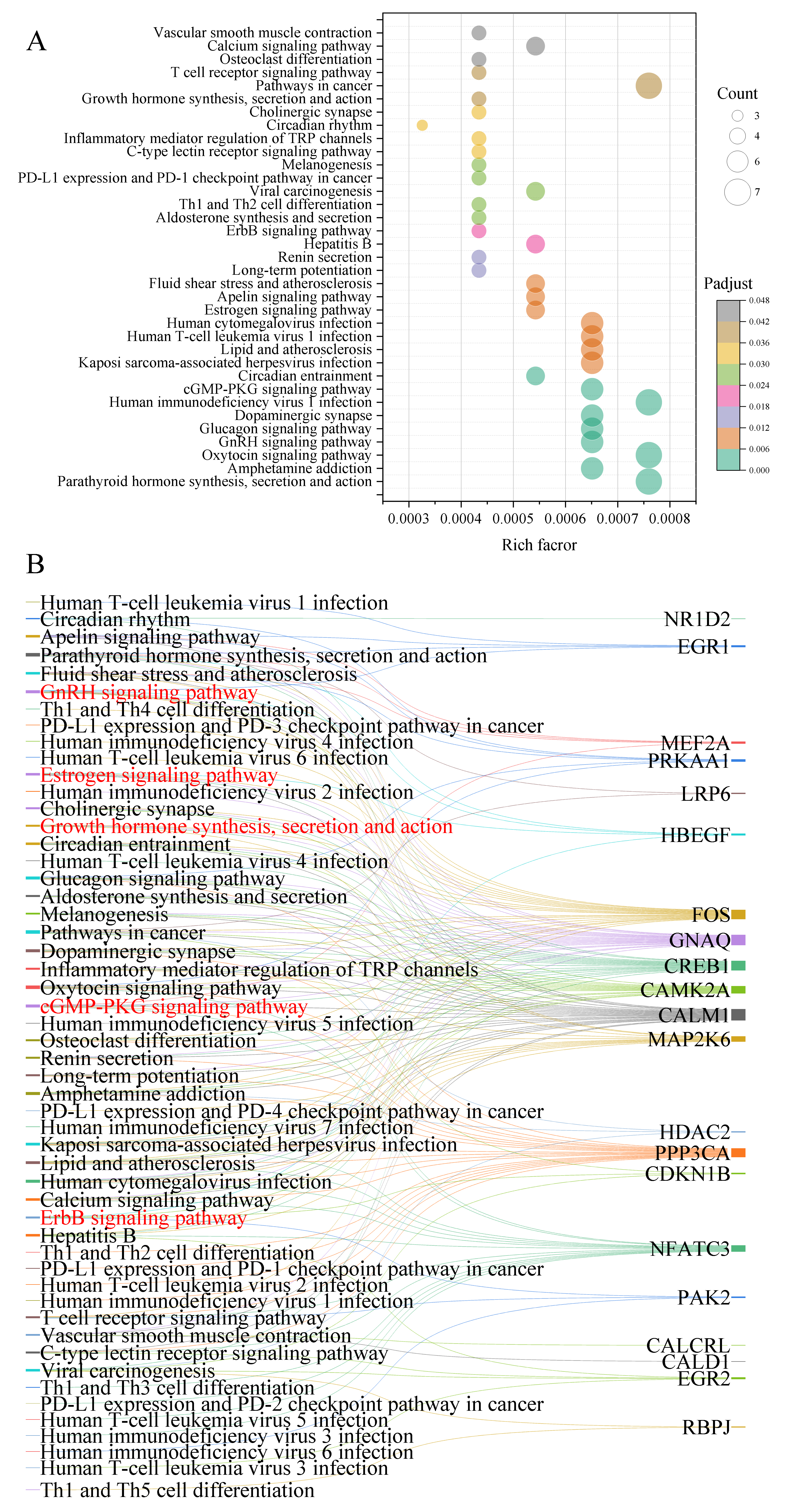

Supplement: Supplementary file 1 [file ijms-25-09089-s001.zip › ijms-3117345-supplementary/Figure S2.tif]

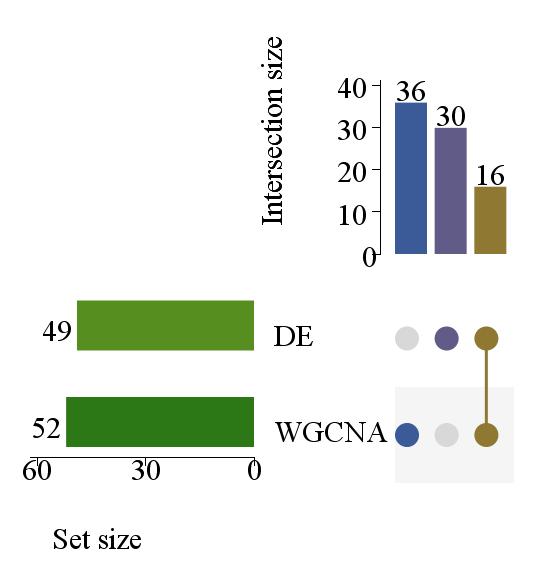

Supplement: Supplementary file 1 [file ijms-25-09089-s001.zip › ijms-3117345-supplementary/Figure S3.jpg]
